# Management of advanced Parkinson’s disease in Israel: Clinicians viewpoint and action items

**DOI:** 10.3389/fnagi.2022.1029824

**Published:** 2022-10-27

**Authors:** Tanya Gurevich, David Arkadir, Samih Badarny, Sandra Benizri, Oren Cohen, Ruth Djaldetti, Sharon Hassin-Baer, Meir Kestenbaum, Zeev Nitsan, Yair Zlotnik, Gilad Yahalom

**Affiliations:** ^1^Movement Disorders Unit, Tel Aviv Medical Center, Sagol School of Neuroscience, Tel Aviv University, Tel Aviv, Israel; ^2^Sackler School of Medicine, Tel Aviv University, Tel Aviv, Israel; ^3^Department of Neurology, Faculty of Medicine, Hadassah Medical Organization, Hebrew University, Jerusalem, Israel; ^4^Galilee Medical Center, Naharyia, Israel; ^5^Azrieli Faculty of Medicine, Bar Ilan University, Safed, Israel; ^6^Movement Disorders Unit, Assuta Ramat HaHayal Hospital, Tel Aviv, Israel; ^7^Department of Neurology, Shamir (Assaf Harofeh) Medical Center, Zerifin, Israel; ^8^Department of Neurology, Movement Disorders Clinic, Rabin Medical Center—Beilinson Hospital, Petach Tikva, Israel; ^9^Department of Neurology, Movement Disorders Institute, Chaim Sheba Medical Center, Ramat-Gan, Israel; ^10^Department of Neurology, Meir Medical Center, Kfar-Saba, Israel; ^11^Movement Disorder Clinic, Barzilai Medical Center, Ashkelon, Israel; ^12^Department of Neurology, Movement Disorders Clinic, Soroka University Medical Center, Beersheba, Israel; ^13^Department of Neurology, Movement Disorders Clinic, Shaare Zedek Medical Center, School of Medicine, Hebrew University, Jerusalem, Israel

**Keywords:** Parkinson’s disease stage-appropriate healthcare facilities, Delphi criteria, burden on public health care systems, clinical challenges, tailored management programs, patient-centered holistic management, intensified device aided therapies, advanced Parkinson’s disease

## Abstract

Parkinson’s disease (PD) is taking a staggering toll on healthcare systems worldwide, with the bulk of the expenditures invested in the late stages of the disease. Considering the rising life expectancy and the increasing prevalence of PD across the globe, a clear understanding of the early signs and treatment options available for advanced PD (APD), will facilitate tailoring management programs and support services. This task is complicated by the lack of both global consensus in defining APD and standardized care guidelines. This perspective prepared by a panel of movement disorder specialists, proposes to extend and optimize currently accepted PD coding to better reflect the diverse disease manifestations, with emphasis on non-motor features. The panel seeks to promote timely diagnosis by adjustment of evaluation tools for use by community neurologists and suggests modification of eligibility criteria for advanced therapy. Moreover, it advocates multidisciplinary assessments of APD patients to drive personalized, patient-centered and holistic management. Overall, earlier and more targeted intervention is expected to markedly improve patient quality of life.

## Introduction

Parkinson’s disease (PD) currently affects 41 in 100,000 individuals between the ages of 40 and 49 and 1,607 in 100,000 individuals over the age of 80 (Pringsheim et al., 2014; Elbaz et al., 2016; Dorsey and Bloem, 2018). In Israel, the prevalence in 2007 was estimated at 256:100,000 (Chillag-Talmor et al., 2011). By 2040, PD is expected to affect approximately 14.2 million individuals worldwide (Dorsey and Bloem, 2018). Its onset is influenced by a host of genetic and environmental factors, with age serving as a central determinant, as well as the most critical risk factor of disease progression and responsiveness to treatment (Levy, 2007; Collier et al., 2011). The disease takes a marked toll on healthcare resources, incurring an estimated $51.9 billion in direct and indirect costs in 2017 in the United States alone (Yang et al., 2020). The largest proportion of expenditures is invested in patients in late stages of the disease, as unidirectional phenotype shifts result in progressive disability and severely compromised patient quality of life (Lim et al., 2009). Given the rising life expectancy across the globe, and increased PD prevalence in the world (GBD 2016 Parkinson’s Disease Collaborators, 2018), PD burden on public healthcare systems is expected to grow, and will require reconsideration of health policies and programs to adequately address the growing needs of the PD population. In this viewpoint, special attention is given to advanced PD (APD), also referred to as complex PD, estimated to impact 10% of the PD patient population (Worth, 2013; Giugni and Okun, 2014).

### Evolution of the clinical picture of Parkinson’s disease

Historically, motor syndrome was the main recognized clinical manifestation of PD and the prevalence of severe disability and mortality within 5 and 10 years of onset was 25 and 65%, respectively (Maier Hoehn, 1992). Yet, since the introduction of levodopa, the mainstay of modern PD treatment, PD-associated motor syndrome has proven responsive to the pharmaceutical treatments and mortality rates have declined, albeit remaining higher than in age-matched controls (Chen et al., 2006). Owing to the remarkable progress in the treatment of motor manifestations, PD is now considered a relatively slowly-progressing, chronic disease with distinctly different manifestations at its various stages, with the APD stage being the most challenging for patients and healthcare providers.

### Advanced Parkinson’s disease: A multisystem disease

**FIGURE 1:**
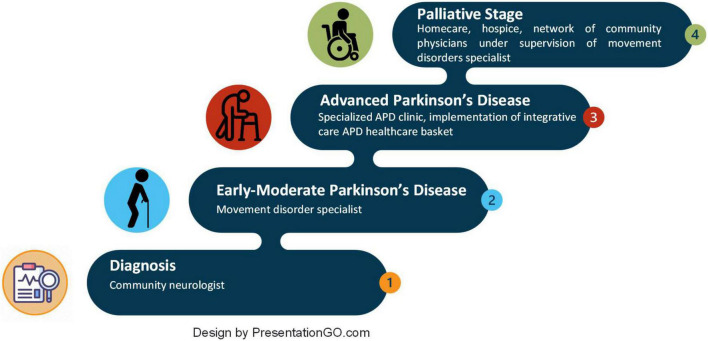
Parkinson’s disease stages and stage-appropriate healthcare facilities.

This stage is characterized by moderate to severe motor deficits (Hoehn & Yahr stage III-V during off periods), generally accompanied by troublesome motor and non-motor symptoms: fluctuations, dyskinesias, frequent off-periods, postural instability leading to frequent falls with increased risk of fractures, sleep disturbances, hallucinations, and cognitive decline, among others. However, with close support, patients are not entirely dependent at this stage and are still capable of independent activity, and may be effectively managed by timely adjustment of the treatment. As PD patients progress to the advanced stage, they typically require intensive and individualized multidisciplinary pharmacological and non-pharmacological care to manage disease and treatment-related complications (Figure 1). Furthermore, as PD symptoms become less controlled with conventional therapies, targeted treatment options, including device-assisted therapies (DAT), such as deep brain stimulation or continuous levodopa-carbidopa infusions *via* pumps, are needed to improve response fluctuations.

### Clinical challenges of advanced Parkinson’s disease

Despite the wealth of knowledge of PD pathology and its natural course, there is a lack of global consensus regarding the precise definition of APD. This is largely due to its heterogenic origins, the vast variety of subphenotypes, variable trajectories and prognostics, as well as the absence of robust biomarkers for disease progression. In turn, it has challenged development of standardized care guidelines, and has brought to a lag in appropriate alignment of diagnostic instruments and grading scales for classification of disease severity and evaluation of treatment and management strategies. Furthermore, it has stymied early APD identification, and tailoring of interventional programs and appropriate allocation of funds. The most popular assessment tools use PD duration as an anchor and primarily focus on cardinal overt motor features. They have been proven to lack universality due to different phenotypes and rates of disease progression and generally fail to recognize the true weight of non-motor features on patient performance and quality of life (Braak et al., 2003; Wolters, 2008). Recent initiatives to develop comprehensive toolkits for evaluating PD status have been steered by the increasing understanding that motor disability, non-motor manifestations, treatment-related complications, and comorbidities are central contributors to APD and its associated limited activities of daily living (ADL), disability and greatly impaired quality of life (Korczyn, 1999; Martinez-Martin et al., 2011; Ray Chaudhuri et al., 2013). All these aspects are well-represented in the recently defined Delphi criteria for APD, which integrate degree of control achieved with oral anti-PD medications, assessment of an array of motor and non-motor symptoms, as well as patient functioning and independence (Antonini et al., 2018). The cross-sectional, multinational, observational OBSERVE-PD review of 2,615 PD patient charts (Fasano et al., 2019) found most significant agreement between physician global assessment and APD diagnosis based on the Delphi criteria, with regards to current treatment programs, limited ADL, motor fluctuations and time from diagnosis. In a subanalysis of the Israeli cohort of 120 patients, physician judgment in classifying APD correlated with select Delphi criteria (Djaldetti et al., 2018). Recently, the intensified therapy component of the “5-2-1” criteria proposed by the Delphi expert consensus panel for identifying APD, has been shown to correlate with established disease burden predictors, including extended disease duration, increased motor and non-motor burden, and compromised quality of life (Fasano et al., 2019; Aldred et al., 2020; Santos-Garcia et al., 2020; Barer et al., 2022) and is included in the recently published MANAGE-PD comprehensive screening tool (Antonini et al., 2019). Integration of wearable sensors into clinical practice are projected to provide objective, quantitative digital patient function-related markers, and thereby improve the sensitivity, accuracy and feasibility of the assessment of motor and non-motor symptoms of PD and diagnosis of APD (Mirelman et al., 2021).

## Discussion

### Definition and diagnosis of advanced Parkinson’s disease

In line with the global efforts to moderate PD impact on quality of life, the authors, representing a panel of Israeli movement disorders specialists, propose to define APD in the International Classification of Disease 11th revision (ICD-11) as a unique health entity that demands adjusted healthcare provider attitudes and relevant social services. Modification of the ICD-11 PD coding should include severity- and fluctuation-based subcodes that accurately capture APD and distinguish it from early-stage PD. While very few diseases have been assigned severity-based subcodes in ICD-10 (diabetes, alcoholic liver disease, renal insufficiency, residual schizophrenia), the growing evidence of the distinct clinical manifestations and medical needs of this patient subpopulation, justifies reconsideration of its coding status.

In addition, we call for optimization of the Delphi criteria by extending them to include a more extensive list of non-motor features, such as autonomic disturbances (e.g., orthostatic hypotension, urinary incontinence), pain, daytime somnolence, and apathy. Furthermore, in the opinion of the Israeli panel, the Delphi criteria for APD from motor fluctuations, regardless of their duration and severity, should be the main eligibility criteria for advanced therapy. Evaluation of PD patients should be based on a structured questionnaire applied as a preliminary tool geared to be implemented by community neurologists or case managers (e.g., nurse practitioners). Patients with suspected APD should undergo multidisciplinary evaluation, ideally in specialized APD centers, to define the extent of disability, outline an individualized treatment program, and weigh the need for rehabilitation and social support services. Routine evaluations should be adequately sensitive to allow for timely diagnosis of palliative-stage PD, which should be addressed by end-of-life palliative/hospice referral (Akbar et al., 2021).

### Comprehensive management of advanced Parkinson’s disease

Management protocols should implement personalized patient-centered and holistic approaches to target the heterogenic manifestations and course of PD. These should include tools for timely diagnosis and treatment of osteoporosis. Furthermore, patients should be informed of opportunities to participate in relevant clinical trials. In addition to the clinical benefits of integrated and coordinated care (Nijkrake et al., 2009; van der Eijk et al., 2011; Loewenbrück et al., 2020; Tenison et al., 2020), such programs have been associated with improved psychological health indicators and self-management capacities (Coulter et al., 2015; Minkman, 2016). Rehabilitation facilities should be staffed by multidisciplinary teams of physiotherapists, speech and swallowing therapists, occupation therapists and social workers. Such programs should be coordinated by specialized nurses (or nurse practitioners) under the supervision of a movement disorders specialist (Cohen et al., 2021).

The panel also suggests standardization of DAT eligibility and prioritization of its use to early-stage APD patients, while minimizing its use in palliative-stage patients. Furthermore, integration of telemedicine and nurse practitioners can tighten surveillance and improve treatment optimization efforts.

These can be further supported by establishment of a network of community physicians, led by movement disorder specialists, to promote case-sharing, research dissemination and exchange of professional know-how.

### Social assistance to patients with advanced Parkinson’s disease

Expanded APD-geared health baskets clearly outline eligibility for reimbursement for a part/full time paid attendant, mobility allowance, and rehabilitation services. In parallel, attention should be paid to informal and non-specialized caregivers by providing them PD-specific education and support (Rosqvist et al., 2021). Patients and caregivers should be made aware of PD-oriented organizations and social networking groups.

## Summary

In summary, the globally rising life expectancy has increased the prevalence of PD in general, and of APD, in particular. Appreciation of the heterogeneity of PD etiology and manifestations has underscored the need for updated PD coding. Precise and standardized definition and evaluation of APD will promote earlier APD identification and timely referral to adequate therapies and specialists. Moreover, it will enhance holistic management, which is expected to markedly improve APD patient quality of life. Future works should focus on validating the proposed extension of the Delphi APD criteria, and on tailoring treatment to APD phenotypes.

## Author contributions

TG: draft preparation and literature search. All authors conceptualized, critically revised, and approved the submitted version.
